# Potential Impact of the Nonessential Energy-Dense Foods Tax on the Prevalence of Overweight and Obesity in Children: A Modeling Study

**DOI:** 10.3389/fpubh.2020.591696

**Published:** 2021-01-28

**Authors:** Daniel Illescas-Zárate, Carolina Batis, Ivonne Ramírez-Silva, Rossana Torres-Álvarez, Juan A. Rivera, Tonatiuh Barrientos-Gutiérrez

**Affiliations:** ^1^Center for Nutrition and Health Research, National Institute of Public Health of Mexico, Cuernavaca, Morelos, Mexico; ^2^CONACYT—Center for Nutrition and Health Research, National Institute of Public Health, Cuernavaca, Morelos, Mexico; ^3^Center for Research in Population Health, National Institute of Public Health of Mexico, Cuernavaca, Morelos, Mexico

**Keywords:** overweight, obesity, energy balance, mathematical model, Mexico, children, taxed food

## Abstract

**Background:** Consumption of foods high in energy, sugar, fat, and salt contributes to the increase in body mass index and the prevalence of overweight and obesity in children. Mexico implemented an 8% tax to non-essential energy-dense foods (NEDF) in 2014 as part of a national strategy to reduce obesity.

**Objective:** We modeled the potential effect of the NEDF tax on body mass index and overweight and obesity in Mexican children (6–17 years).

**Materials and Methods:** We used the Dynamic Childhood Growth and Obesity Model calibrated to Mexican children to simulate the potential 1-year effect of the NEDF tax on body weight. Inputs for the model included NEDF consumption, weight, and height, obtained from the 2012 Mexican National Health and Nutrition Survey. To project the potential impact of the tax, we ran a first simulation without intervention and another reducing the caloric intake from NEDF in the proportion observed in the Mexican population after the tax (−5.1%). The tax effect was defined as the absolute difference in body mass index and prevalence of overweight and obesity between both models.

**Results:** The tax on NEDF should lead to a mean reduction of 4.1 g or 17.4 kcal/day of NEDF at the population level. One year after the tax, mean body weight and body mass index should decrease 0.40 kg and 0.19 kg/m^2^; this translates into −1.7 and −0.4% points in overweight and obesity, respectively.

**Conclusions:** The use of fiscal instruments to discourage the consumption of NEDF could help to reduce the prevalence of overweight and obesity in children.

## Introduction

Childhood obesity is caused by poor regulation of energy balance over a long period of time, in which energy consumption is higher than a child's energy expenditure and normal body growth (1–3). The prevalence of obesity in school-aged children and adolescents in Mexico is among the highest in the world (4–6). The rapid increase in childhood obesity is a major public health concern, leading to the development and implementation of structural interventions to improve diet (7, 8).

Consumption of non-essential energy-dense foods (NEDF) is considered to be a main driver of obesity (9). NEDF are high in added sugars, total fat, energy, and salt, can contribute to a significant proportion of the daily energy intake, and have been associated with weight gain (10, 11). To disincentivize their consumption, countries like Hungary, Denmark, and Australia recently established taxes to non-essential energy-dense foods (12).

In January 2014, Mexico passed an 8% tax to non-essential foods with an energy density exceeding 275 kcal/100 g (13); these foods contribute to 19.7 and 17.9% of the daily energy consumed by school-aged children and adolescents, respectively (14). One year after the implementation, a study estimated that the tax reduced NEDF household purchases by 5.1% (15). However, to date, no study has analyzed the potential impact of this reduction in body weight in children.

We aimed to estimate the potential effect of the NEDF tax on body mass index (BMI) and the prevalence of overweight and obesity in Mexican children, using a mathematical model of childhood energy balance and body weight dynamics to project the expected weight change 1 year after the implementation of the NEDF tax.

## Methods

### Simulation Strategy

**Figure 1:**
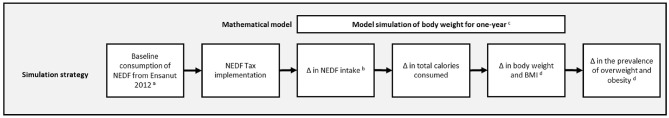
Schematic illustration of the 1-year simulation strategy to estimate the potential effect of the NEDF tax on BMI and the prevalence of overweight and obesity in children aged 6–17 years. ^a^Inputs for the model included Nonessential Energy-Dense Foods (NEDF) consumption, weight, and height, obtained from the 2012 Mexican National Health and Nutrition Survey (ENSANUT 2012) (16). ^b^Change of NEDF consumption from ENSANUT 2012 was estimated at the individual level using an average of 5.1% reduction (15). ^c^Predicted body weight 1 year after the NEDF tax was simulated using the Dynamic Childhood Growth and Obesity model (DCGO) from Hall et al. (17). ^d^Body mass index (BMI) and BMI-z-scores 1 year after the NEDF tax were obtained using the World Health Organization reference to estimate prevalences of overweight and obesity in children (18). We assume that all the change in body weight occurs via reduction in NEDF consumption.

Figure 1 summarizes the simulation strategy used. First, we obtained data of the pre-tax consumption of NEDF, age, sex, weight, and height from a representative sample of Mexican children and adolescents. Then, we calculated the change in NEDF consumption attributable to the tax for each child, assuming that observed changes in purchases translate into the same reductions in individual level consumption; expected changes in NEDF intake in grams were translated into calories. We then used a dynamic weight change model to estimate the 1-year weight change for each child assuming no intervention and an alternative model assuming the NEDF caloric reduction. The difference between scenarios is the projected change in body weight, body mass index, and overweight and obesity prevalence.

### Data Source

Dietary and anthropometric information was obtained from the National Survey of Health and Nutrition 2012 (ENSANUT 2012, for its acronym in Spanish), which was conducted between October 2011 and May 2012 (16). ENSANUT is representative of the Mexican population at the national, regional, urban/rural, and state levels (16, 19). Trained interviewers conducted a 24-h recall (24HR) in a random subsample (~11% of the ENSANUT respondents) and anthropometry in 10,886 subjects. Of this sample, we used information from two subgroups (*n* = 4,140): school-aged children (6–11 years) and adolescents (12–17 years). Pregnant and/or lactating women were excluded from the analysis (*n* = 8).

### Anthropometry

Body weight and height were obtained by trained and standardized personnel according to the procedures and protocols recommended for children (20, 21). We calculated BMI and excluded observations with implausible values (<10 or >38 kg/m^2^ for school-aged children and <10 or >58 kg/m^2^ for adolescents). BMI-for-age and height-for-age *z*-scores were estimated using the AnthroPlus software, as recommended by the World Health Organization (22). We considered plausible BMI-for-age *z*-scores between −5.0 and +5.0 standard deviations and between −6 or +6 standard deviations for height-for-age *z*-score. Four participants were excluded due to these criteria, as well as 153 who did not have anthropometric information.

The prevalence of overweight and obesity in school-aged children and adolescents were defined based on the World Health Organization criteria. Overweight was defined as BMI-for-age *z*-score >1.0 standard deviations and obesity as >2.0 standard deviations (18).

### Dietary Assessment

Dietary information was collected using a 24HR proportionally distributed in the sample across all days of the week, including weekends (23). 24HR automated multiple-pass method was applied following the procedure recommended by the United States Department of Agriculture to minimize underreporting and improve the accuracy of recall (24). The mother or the person in charge of food preparation in the household was responsible for indicating the food consumed by children aged 15 or younger. Children older than 15 years were asked directly about their intakes. Dietary information was collected as (1) single foods such as the consumption of fruit, (2) custom recipes (characterized by the reported preparations where the study subject knew about the food and amounts used by participants), and (3) standard recipes (set of default ingredients that make up a recipe when the informer did not provide one). All recipes were disaggregated into their ingredients (except beverages) to facilitate identifying all NEDF in a recipe (e.g., chips, puffed wheat snacks, candies, chocolate, sweets, others) (23).

NEDF consumption was classified using the definition by the Ministry of Finance in conjunction with the Ministry of Health of Mexico. To be included in this classification, foods needed to comply with two conditions: an energy density ≥275 kcal/100 g and to not to be staple foods for the Mexican population, such as tortillas, toasts, whole grains, other cereals, and non-sweet bread. Food groups in this classification include French fries, salty snacks, cakes, chocolates, candies, sweet bread, frozen desserts, non-cereal based sweets, and ready-to-eat cereals (13). Grams and energy of total consumption were calculated for each subject using the food composition base compiled by the National Institute of Public Health of Mexico (25).

We excluded 55 subjects with extreme values of energy consumption (exceeding ±3 standard deviations of the logarithm of the total energy requirement/total energy consumption ratio) and eight participants without diet information. Our analytical sample included 3,912 school-aged children and adolescents.

### Covariates

Demographic variables such as sex, age, and socioeconomic status (SES) were obtained from ENSANUT 2012 (16). A socioeconomic status index was constructed using principal component analysis that included information on household characteristics and assets (26). The index was then divided using tertiles, to classify participants into low, medium, and high SES.

### Mathematical Model

Dietary and anthropometric information from ENSANUT 2012 were used as inputs for the Dynamic Childhood Growth and Obesity Model (DCGO) proposed and validated by Hall et al. (17). Briefly, the DCGO model is a dynamic weight change model that uses a system of differential equations to predict changes in body weight over time, as energy intake or physical activity vary. An overview of the model and its calibration to the Mexican population by nutritional status is presented in the Supplementary Material. The DCGO model was designed to differentiate healthy growth between male and female individuals, considering the increase in energy consumption as age progresses and the physiological processes that stimulate the synthesis of fat free mass during childhood. As part of energy expenditure, it is assumed that physical activity remains constant from 6 to 12 years old; thereafter, physical activity decreases progressively as age increases (17).

### Change in NEDF Consumption Due to the Tax

To estimate the reduction in consumption of NEDF, which is used as an input for the DCGO model, three studies were used as a reference. These studies evaluated the mean decrease in NEDF purchases in the first 3 years after the tax was implemented (2014, 2015, or 2016), which estimated a 5.1% reduction in purchases of NEDF for the first year (2014 vs. 2013 and 2012) (15) and 6.0% for the first 2 years (2014 and 2015 vs. 2012) (27). The third study was an evaluation performed using the National Survey of Household Income and Expenditure of Mexico in 2014 and 2016, where a mean reduction of 5.3% in NEDF purchase was observed (28).

To maintain a conservative scenario, we assumed that the 5.1% decrease in purchases observed in the first year of the tax translated into the same reduction in NEDF consumption at the individual level. To capture differences by SES, we used the effects stratified as provided in the manuscript: 10.2, 5.8, and 0% for low, medium, and high SES, respectively (15). To translate this reduction into our sample, we first calculated the total grams of NEDF consumption as reported for each child; then, we multiplied it by the percent reduction that corresponded to his/her SES. Subsequently, the change in grams of NEDF was transformed into calories, assuming a mean NEDF energy density of 430 kcal/100 g, as previously estimated (15). Thus, for instance, for a low-SES child who consumed 30 g of NEDF at baseline, the tax is expected to decrease 3.06 g of NEDF, equivalent to 13.16 kcal.

### Model Specification

The DCGO model was used to obtain two scenarios of body weight in the sample with a 1-year simulation time frame. The first scenario assumed no intervention. The second scenario was simulated using the same inputs as scenario 1 but under the effect of the NEDF tax. To estimate BMI at the end of the modeling year, we used the body weight estimated by the model and projected the growth in height assuming that each child maintained the same height-for-age *z*-score. The prevalence of overweight and obesity were estimated using the BMI-for-age *z*-score. The expected impact of the NEDF tax was defined as the absolute difference between the model under the tax and the model without intervention 1 year after implementation. This estimate assumes that calories reduced by the NEDF tax are not compensated by other foods; as a sensitivity analysis, we used 10 and 20% energy compensation.

### Statistical Analysis

We estimated calories per capita from NEDF and the percentage of consumption that these foods contribute to total energy consumption. In addition, reduction in grams and energy of NEDF attributed to tax implementation was estimated. These variables were described with mean and standard error according to age, sex, and SES.

The expected impact of NEDF tax in body weight, BMI, BMI-for-age *z*-score, and in the prevalence of overweight and obesity was calculated at the national level, by groups of age, sex, and SES. Body weight, BMI, and BMI-for-age *z*-score were described with means and standard error and prevalence as percent and 95% confidence intervals. The DCGO model does not produce confidence intervals (CIs) to the estimators; hence, our CIs were constructed using the error captured by the survey data.

Data processing of demographic, anthropometric, and dietary information, as well as analyses considering the survey design and sampling weights were performed using Stata, version 13 (*Stata Statistical Software; StataCorp*) (29). The DCGO model was implemented using the Dynamic Body Weight Model Package for Children and Adults in R software (30).

## Results

Table 1 shows the characteristics of selected children from ENSANUT 2012. We included 3,912 participants, representing 27.9 million children between 5 and 17 years old in Mexico. The mean consumption of NEDF was 355 kcal/day/capita or 17.7% of total energy consumption. The expected NEDF reduction in consumption as a result of the tax would translate into a mean decrease of 4.1 g or 17.4 kcal per child per day. Table 2 shows that reductions in NEDF calories after the tax should be larger in low and medium SES than in high SES (28.1, 21.3 vs. 0 kcal, respectively), in adolescents than in school-aged children (18.5 vs. 16.7 kcal), and in male than in female children (18.0 vs. 16.8 kcal).

**Table 1:** Characteristics of children 6–17 years of age in Mexico; 2012 Mexican National Survey of Health and Nutrition (ENSANUT 2012).

	**Total sample**		
	****n****	N^*^	**Percentage/mean (SE)**
**SEX (%)**			
Male	1,974	14,025	50.2
Female	1,938	13,890	49.7
**AGE**			
Mean (SE), years	3,912	27,916	10.8 ± 0.07
School-aged children (%)	2,590	17,389	62.4
Adolescent (%)	1,322	10,527	37.6
**AREA (%)**			
Urban	2,386	19,407	69.5
Rural	1,526	8,508	30.4
**SOCIOECONOMIC STATUS (%)**			
Low	1,452	9,288	33.2
Medium	1,376	9,533	34.1
High	1,084	9,094	32.5
**Weight (kg)**	3,912	27,916	39.4 ± 0.40
**Height (cm)**			139.2 ± 0.43
**BMI (kg/m**^**2**^**)**			19.4 ± 0.11
**BMI for Age**, ***z*****-score**			0.58 ± 0.03
**Height for Age**, ***z*****-score**			−0.48 ± 0.02

*Results are presented in percentages or mean ± standard error*.

* *In thousands*.

*BMI, body mass index*.

**Table 2:** Non-essential energy-dense foods consumption and estimated impact of tax according to demographic characteristics in Mexican children aged 6–17 years according to ENSANUT 2012.

	**Sample**		**Total energy consumption^a^**	**NEDF consumption^a^**		**Mean reduction due NEDF tax**	
	***n***	N^*^	**Calories per capita**	**Calories per capita**	**% of total calories**	**Grams^a^**	**Calories^b^**
**Total**	3,912	27,916	1,927 ± 21	355 ± 10	17.7 ± 0.4	4.1 ± 0.1	17.4 ± 0.5
**AGE GROUPS**							
School-aged children	2,590	17,389	1,834 ± 24	341 ± 11	17.5 ± 0.4	3.9 ± 0.1	16.7 ± 0.5
Adolescent	1,322	10,527	2,079 ± 34	378 ± 15	17.9 ± 0.7	4.3 ± 0.2	18.5 ± 0.8
**SEX**							
Male	1,974	14,025	2,032 ± 31	368 ± 12	17.3 ± 0.6	4.2 ± 0.2	18.0 ± 0.6
Female	1,938	13,890	1,820 ± 25	342 ± 13	17.9 ± 0.6	3.9 ± 0.2	16.8 ± 0.6
**SOCIOECONOMIC STATUS**							
Low	1,452	9,288	1,882 ± 34	285 ± 12	15.1 ± 0.6	6.5 ± 0.2	28.1 ± 0.6
Medium	1,376	9,533	1,960 ± 34	383 ± 16	18.6 ± 0.7	4.9 ± 0.2	21.3 ± 0.7
High	1,084	9,094	1,936 ± 37	397 ± 19	19.2 ± 0.8	0	0

*Results are presented in mean ± standard error*.

* *In thousands*.

a *Information about consumption and demographic characteristics of this table were obtained from the 2012 Mexican National Health and Nutrition Survey (ENSANUT 2012) (16)*.

b *Calories estimated from assuming a mean energy density of the NEDF of 430 kcal/100 g*.

*NEDF, non-essential energy-dense foods*.

**Figure 2:**
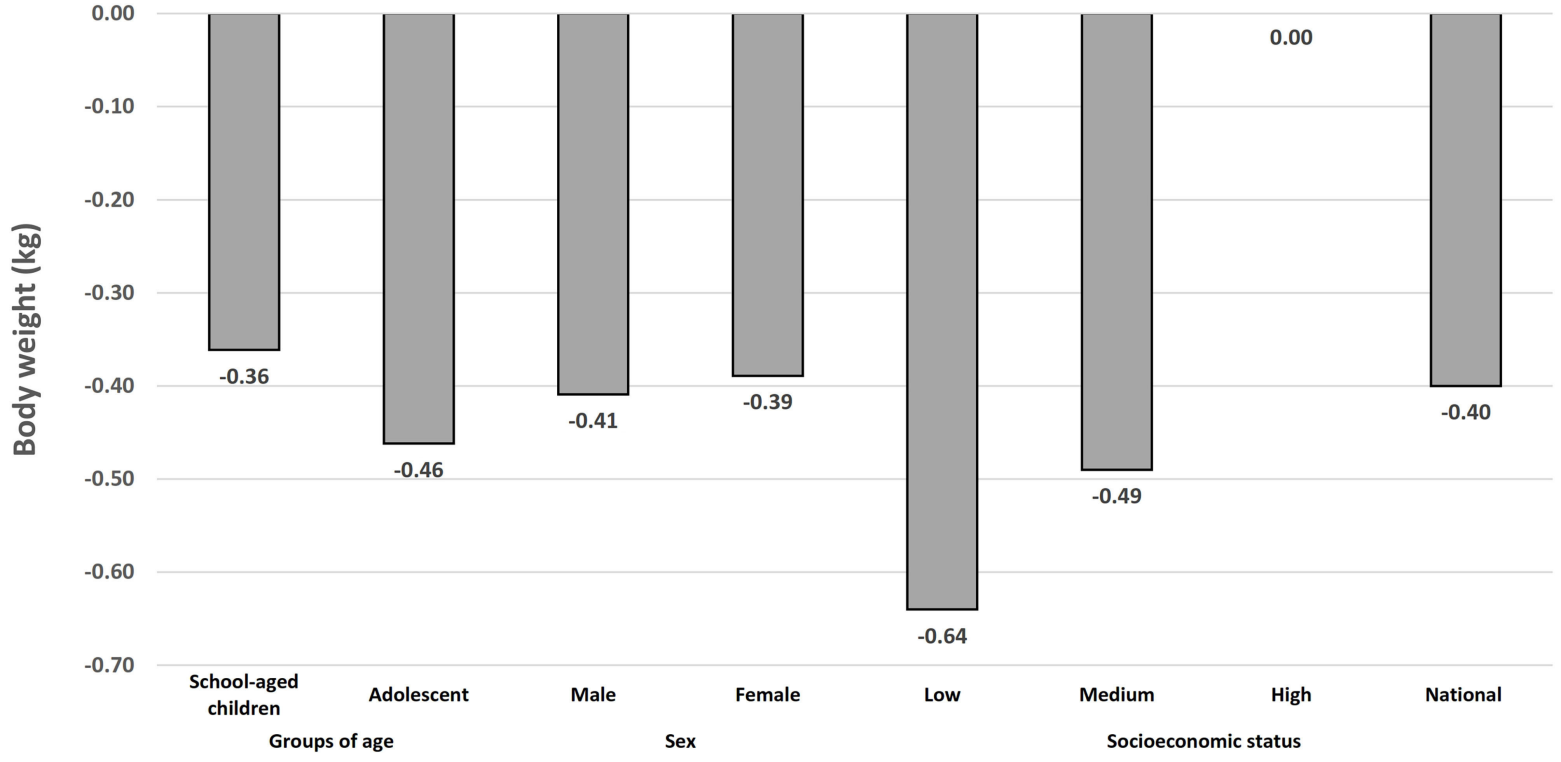
Potential 1-year effect of the Non-essential Energy-Dense Food tax on body weight (kg) in Mexican school-aged children (6–11 years) and adolescents (12–17 years) using the Dynamic Childhood Growth and Obesity Model (*n* = 3,912).^a,b^. ^a^The Dynamic Childhood Growth and Obesity Model (DCGO) was used to obtain two scenarios of body weight in the sample with a one-year simulation time frame. The first scenario assumed no intervention. The second scenario was simulated using the same inputs as scenario 1, but under the effect of the NEDF tax. Absolut differences between both scenarios is the potential effect of NEDF tax on body weight (kg) at the end of the simulation year. ^b^Simulated body weight 1 year after implementation of the NEDF tax, assuming average reduction of 5.1% in Nonessential Energy-Dense Food (NEDF) consumption at the individual level. Differences by socioeconomical status, assumed reductions of 10.2%, 5.8%, and 0% for low, medium, and high SES, respectively (15).

Figure 2 shows the difference in body weight at the end of the simulation year, by sociodemographic characteristics. The tax is expected to reduce −0.40 kg of body weight and −0.19 kg/m^2^ of BMI. Children with low SES (−0.64 kg), adolescents (−0.46 kg), and male children (−0.41 kg) are expected to experience the largest reductions in body weight.

Table 3 shows the expected changes in the prevalence of overweight and obesity in Mexican children after the implementation of the NEDF tax. One year after the tax, the prevalence of overweight should decrease −1.7 pp (95% CI: −0.8, −2.5), while obesity should decrease −0.4 pp (95% CI: −0.1, −0.6). Larger reductions in the prevalence of overweight should occur in school-aged children [−1.8 pp (95% CI: −0.8, −2.7)], male children [−1.9 pp (95% CI: −0.5, −3.1)], and low SES children [−1.9 pp (95% CI: −0.80, −2.9)]. However, the largest impact of the tax should be experienced by children in the medium SES group, with a 2.6 pp reduction in overweight (95% CI: −0.60, −4.6). Reductions in the prevalence of obesity should be larger in school-aged children [−0.4 pp (95% CI: −0.1, −0.8)], male children [−0.5 pp (95% CI: −0.1, −0.9)], and in children in the low SES group [−0.6 pp (95% CI: −0.01, −1.0)]. Point estimates and 95% confidence intervals for expected changes in BMI and BMI-for-age *z*-score are available in Supplementary Table 1.

**Table 3:** Potential impact 1 year after the implementation of the NEDF tax on overweight and obesity prevalence by sociodemographic characteristics using the Dynamic Childhood Growth and Obesity Model^a^.

	**Overweight**		**Obesity**		
	**Without tax [% (95% CI)]**	**With tax [% (95% CI)]**	**Absolute difference (pp)**	**Without tax [% (95% CI)]**	**With tax [% (95% CI)]**	**Absolute difference (pp)**
**Total**	21.2 (19,23)	19.4 (18,22)	−1.7 (−0.80,−2.5)	13.8 (12,15)	13.4 (11,15)	−0.4 (−0.10,−0.60)
**AGE GROUPS**						
School-aged children	21.5 (19, 24)	19.6 (17, 22)	−1.8 (−0.80, −2.7)	13.6 (12, 16)	13.1 (11, 15)	−0.4 (−0.10, −0.80)
Adolescent	20.7 (18, 24)	19.1 (16, 22)	−1.5 (−0.10, −3.0)	14.1 (11, 18)	13.9 (11, 18)	−0.2 (−0.10, −0.40)
**SEX**						
Male	20.6 (18, 24)	18.7 (16, 22)	−1.9 (−0.50, −3.1)	16.2 (13, 19)	15.6 (13, 18)	−0.5 (−0.10, −0.90)
Female	21.7 (19, 25)	20.2 (17, 23)	−1.5 (−0.40, −2.4)	11.4 (9.6, 13)	11.2 (9, 13)	−0.2 (−0.10, −0.40)
**SOCIOECONOMIC STATUS**^**b**^						
Low	23.7 (20, 27)	22.1 (19, 26)	−1.9 (−0.80, −2.9)	11.3 (9, 14)	10.8 (9, 13)	−0.6 (−0.01, −1.0)
Medium	20.3 (17, 24)	17.7 (15, 21)	−2.6 (−0.60, −4.6)	13.7 (11, 17)	13.2 (11, 16)	−0.5 (−0.12, −0.96)
High	19.4 (16, 23)	19.4 (16, 23)	0.0 (0.0, 0.0)	16.1 (13, 19)	16.1 (13, 19)	0.0 (0.0, 0.0)

a *The Dynamic Childhood Growth and Obesity Model (DCGO) was used to obtain a no intervention (without tax) and a tax scenario (with tax) for body weight using a 1-year simulation time frame; the absolute difference is the expected impact of the NEDF tax on overweight and obesity*.

b *Body weight simulated under the tax, assumed an average reduction of 5.1% in non-essential energy-dense food (NEDF) consumption at the individual level. To obtain differences by SES, we used the effects stratified as follows: 10.2, 5.8, and 0% for low, medium, and high SES, respectively (15)*.

*BMI, body mass index; NEDF, non-essential energy-dense foods; SE, standard error; pp, percentage points*.

Table 4 shows 1-year-simulation results of the sensitivity analysis, testing the potential effect of tax on NEDF assuming a 10 and 20% energy compensation. In these models, we found a slight attenuation of the expected impact in BMI, BMI-for-age *z*-score, and overweight prevalence but a similar reduction in the prevalence of obesity than without dietary compensation.

**Table 4:** Sensitivity analyses for the potential 1-year effect of the NEDF tax on BMI and overweight and obesity, based on 10 or 20% energy intake compensation (*n* = 3,912).

**Simulated scenarios of the NEDF tax effect**	**BMI (mean ± SE)**	**BMI for age *z*-score (mean ± SE)**	**Overweight [pp (95% CI)]**	**Obesity [pp (95% CI)]**
**Absolute difference**^a^ **without energy compensation**	−0.19 ± 0.01	−0.09 ± 0.01	−1.7 (−0.80, −2.5)	−0.4 (−0.10, −0.60)
**Dietary energy compensation**
Absolute difference^a^ with 10% of energy compensation	−0.17 ± 0.01	−0.08 ± 0.01	−1.4 (−0.60, −2.1)	−0.3 (−0.10, −0.50)
Absolute difference^a^ with 20% of energy compensation	−0.15 ± 0.01	−0.7 ± 0.01	−1.3 (−0.60, −2.1)	−0.3 (−0.10, −0.50)

*We assume that all the change in body weight occurs via reduction in NEDF consumption*.

a *Absolute difference between the no intervention scenario and the NEDF tax scenario. Difference calculated 1 year after the implementation of the tax*.

*BMI, body mass index; NEDF, non-essential energy-dense foods; SE, Standard error; pp, percentage points*.

## Discussion

We modeled the potential effect of the 8% NEDF tax in Mexico on body weight and the prevalence of overweight and obesity in children between 5 and 17 years old. According to our estimates, the observed reductions in NEDF consumption after the tax should result in a national average reduction in energy consumption of 17.4 kcal/person/day and a weight difference of −0.40 kg, −0.39 kg/m^2^ in BMI, and a −1.7 and a −0.4 pp in the prevalence of overweight and obesity at the end of the first year with the tax. Children in the low and middle SES groups will benefit from the tax, although the middle group will experience a larger difference. Children in the high SES group are not expected to benefit from this policy, given that no reduction in NEDF consumption has been observed in this group (15).

NEDF consumption is an important source of energy in Mexican children that has been proven to be reduced with the NEDF tax. NEDF are large contributors to the total energy consumption in Mexican children between 5 and 19 years old, with an estimated 18.8% of the daily caloric intake (14). An international comparison using the tax-based NEDF definition is difficult, but those NEDF are a subgroup of ultraprocessed foods according to the NOVA classification. In Mexico, school-aged children consume 34.3% of their total energy intake from ultraprocessed foods, while adolescents consume 35.5% (31). In contrast, in 2005, Colombian children aged 2–9 years consume 18.5% of total energy intake, while adolescents consume 18.6% (32); in 2010, Chilean children 2–19 years consumed 37.6% of their total energy intake from ultraprocessed foods (33). In contrast, in Canada and in the US, ultraprocessed foods represent 55 and 65% of the total energy intake, respectively (33–35). The tax in Mexico was observed to reduce NEDF consumption at least 5.1%, which should lead to a reduction of 0.39 kg/m^2^ in BMI and 0.09 fewer units in BMI-for-age *z*-score. This could seem small from an individual perspective, but at a population level, it is considerable. Moreover, results should be interpreted considering that we only modeled 1 year after implementation and that changes over time could extend beyond this period.

The NEDF tax represents an 8% price increase, which, compared to sugar-sweetened beverages taxes (10%), is low and very low if compared to the 66.9% price increase produced by tobacco taxes in Mexico (7, 36). Tobacco taxes are an excellent example of the potential of taxes to reduce the use of harmful products; in tobacco control, adolescents have proven to be more responsive to taxes than adults, reducing 4% in cigarettes consumed for each 10% increase in price (36–38).

Achieving a reduction in the consumption of NEDF in children is an important objective to improve children's health. Lower NEDF consumption implies a significant improvement in diets, as NEDF are rich in added sugar, saturated fat, and sodium, which have been associated with obesity in children (33–35, 39, 40). Body weight loss in children has been linked with improvement in blood pressure, low-density lipoprotein (LDL)-cholesterol, high-density lipoprotein (HDL)-cholesterol, triglycerides, or insulin resistance even if the BMI *z*-score reduction is small (41); thus, small but sustained weight loss or prevention of unhealthy weight gain could help reverse the burden of disease that derives from obesity in children.

To our knowledge, this is the first study to estimate the potential impact of the NEDF tax on body weight, BMI, and overweight and obesity prevalence in children. Consequently, we could not identify studies directly comparable to ours. However, to put our estimates into perspective, we will compare them with the effects achieved by randomized trials to reduce weight in children and with other simulation models. Empirical studies in children 2–18 years old, focusing on dietary improvement, have achieved reductions in BMI-for-age *z*-score of −0.04 (95% CI: −0.08 to −0.01) (42) and up to −0.09 (95% CI: −0.13 to −0.05), which are within the range of our estimates as presented in Supplementary Table 1 (43). Our results are also within the range of effects estimated by previous modeling studies for other food policies in children. Brown et al., using the same mathematical model, estimated the potential effect of reducing 27 kcal/day associated to restrictions to TV advertising of beverages and foods in children 5–15 years of age, which was expected to reduce 0.35 kg/m^2^ of BMI within 1 year (44); in contrast, we estimated a 0.19 kg/m^2^ reduction from the 17.4 kcal/day reduction expected from the NEDF tax. Torres-Alvarez et al. used the same model to estimate the impact of the sugar-sweetened beverages tax in Mexican children 5–17 years old; the 10% tax is expected to reduce 17.6 kcal/day, which should translate into a 0.42 kg body weight reduction (45).

The prevalence of overweight and obesity in school-aged children and adolescents is very high in Mexico (46). We estimated that the national prevalence of overweight and obesity effect should be higher in school-aged children, male children, and children in the low SES group. School-aged and male children are the groups with the highest NEDF intake among all children, which explains why the tax could produce larger decreases in these groups (14). In contrast, the larger reduction in body weight observed in low SES is related to a stronger response of this group to the NEDF tax, as observed by Batis et al. (15). While the estimated reduction in overweight and obesity is small, the prevention of obesity and overweight cases in this age group could lead to large improvements in cardiometabolic health later in life, considering that childhood obesity is a strong predictor of obesity and chronic diseases (47, 48). This is an important finding that points to the equitability of the NEDF tax, as children in the lowest quintile of SES in Mexico are experiencing the highest increases in overweight and obesity and will carry the heaviest burden of chronic diseases in the future (46). In that sense, the NEDF tax is a progressive measure, as it will likely reduce future healthcare costs for the less favored individuals (7). Given our estimates, it is evident that increases to the NEDF tax will be needed to produce larger reductions in the prevalence and that fiscal measures will need to be accompanied by other population strategies to help reduce overweight and obesity in children.

In ENSANUT 2012, it was observed that the mean energy consumption exceeds the total energy requirement by 10% in school-aged children and up to 7% in adolescents (23). This translates into an average imbalance of 190 and 165 extra kcal/day, respectively (17, 49). In the present study, energy reduction due to NEDF tax was estimated at 16.7 kcal in school-aged children and 18.5 kcal in adolescents, which provides a quantitative estimate of the magnitude of the interventions needed to prevent obesity in future generations. Sugar-sweetened beverages tax is another intervention in Mexico designed to reduce energy consumption in the population, and the potential effect estimated was 11.7 kcal/day in school-aged children and 24.4 kcal/day in adolescents. These two interventions encompass just 15.0 and 26.0% of the excess in energy consumption in these groups of age. Therefore, none of these two interventions by itself would be sufficient to reverse the obesity trend in Mexico. Instead, a comprehensive strategy including effective interventions covering from pregnancy to adulthood to prevent excessive weight gain is needed (50). For instance, Australia has proposed a national strategy to reduce the obesity prevalence by increasing breastfeeding and physical activity, improving the food environment, and reducing the consumption of unhealthy foods through taxes, while increasing the consumption of healthy foods through subsidies (51).

Our study has some limitations that must be considered. First, the expected change in NEDF intake due to the tax was obtained from an observational study that estimated the impact of the tax in NEDF purchases (15); thus, we had to assume that the observed changes in household purchases translate into changes in individual-level intake. This assumption is common to prior studies that have estimated the impact of sugar-sweetened beverages on body weight (52–55). We used the 5.1% decrease in NEDF household purchases reported by Batis et al. as a conservative estimate, although a subsequent evaluation observed a mean reduction in NEDF purchases of 6.0% in 2014–2015, which is related to a larger reduction in purchases in the second year of tax (27). Therefore, the estimated effect on body weight and the prevalence of overweight and obesity in our study could be underestimated. Second, the model assumes a steady state, that is, it assumes that obesity rates are not increasing and that no other intervention affects the outcome. Third, we assume food compensation of 10 and 20% for a reduction in NEDF consumption, which implies that at least 80% of the effect of the intervention translates into a child's total energy expenditure and, subsequently, to body weight. There is no consensus on the compensatory effect for dietary energy reduction, especially in the long term (56). However, a recent study reduced 8% of energy density in preschoolers' diet (72 kcal/day) and maintained the gap without energy compensation during the trial (5 days) (57). This suggests that the reduction in NEDF consumption given the implementation of tax might have a similar effect to this study, in which energy reduction is maintained over time.

In conclusion, our study suggests that the NEDF tax should reduce overweight and obesity in children 5–17 years old. Our analysis by SES demonstrates a greater impact on those living in socioeconomic disadvantage compared to least disadvantaged children. The use of fiscal instruments to discourage consumption of NEDF is a strategy to tackle childhood obesity that should be considered an integral part of any overarching strategy to reduce childhood obesity. The current 8% tax should be increased to produce larger health gains.

## Data Availability Statement

The model developed by Hall et al. (58) and used for this study is available in R (http://github.com/INSP-RH/bw-Mexican-children-population). Data from ENSANUT 2012 used as inputs for the model are publicly available in www.ensanut.insp.mx (16, 17).

## Ethics Statement

A signed assent form and signed parent/guardians consent form were required to obtain children's measurements and participate in ENSANUT 2012. This study was reviewed and approved by the ethics and research committees of the National Institute of Public Health of Mexico.

## Author Contributions

DI-Z and TB-G designed the study. RT-Á contributed to the model development and conducted the model simulations. DI-Z wrote the first draft of the manuscript and analyzed the data. All authors review model parameters, inputs, and provided important intellectual contributions to the revision of the manuscript and approval of the final manuscript.

## Conflict of Interest

The authors declare that the research was conducted in the absence of any commercial or financial relationships that could be construed as a potential conflict of interest.

## Abbreviations

NEDF: non-essential energy-dense foods
BMI: body mass index
ENSANUT 2012: Mexican National Health and Nutrition Survey 2012
24HR: 24-h recall
SES: socioeconomic status
DCGO: Dynamic Childhood Growth and Obesity Model
FM: fat mass
FFM: free fat mass
CIs: confidence intervals.
