# Supplementary material for: Potential Impact of the Nonessential Energy-Dense Foods Tax on the Prevalence of Overweight and Obesity in Children: A Modeling Study
Source: Front Public Health. 2021 Jan 28;8:591696. doi: 10.3389/fpubh.2020.591696 (PMC7902009; doi:10.3389/fpubh.2020.591696)
Supplement: Supplementary file 1 [file Data_Sheet_1.PDF]

Supplementary material

Potential Impact of the Nonessential Energy-Dense Foods Tax on the Prevalence of Overweight and Obesity in Children: A modeling study

Daniel Illescas-Zárate[1], Carolina Batis[2], Ivonne Ramírez-Silva[1], Rossana Torres-Álvarez[3], Juan A. Rivera[1], and Tonatiuh Barrientos-Gutiérrez[3*].

[1] Center for Nutrition and Health Research, National Institute of Public Health of Mexico, Cuernavaca, Morelos, México.
[2] CONACYT – Center for Nutrition and Health Research, National Institute of Public Health, Cuernavaca, Morelos, México.
[3] Center for Research in Population Health, National Institute of Public Health of Mexico, Cuernavaca, Morelos, México.

*Correspondence
Tonatiuh Barrientos-Gutiérrez
tbarrientos@insp.mx

Table of Contents

Supplementary table 1. Potential impact one year after the implementation of the NEDF tax on body mass index and body mass index-for-age z-score using the Dynamic Childhood Growth and Obesity Model........ 3

Supplementary weight change model specification........ 4

Childhood Growth and Obesity Model........ 4

Model simulations........ 4

Dynamics of Childhood Growth and Obesity Model: validation according to nutritional status........ 6

Supplementary figure 1. Comparison of mean body weight between the Dynamics of Childhood Growth and Obesity Model and mean observed values in Ensanut 2012 according to nutritional status in children from 5 to 17 years old........ 6

Supplementary Figure 2. Comparison of mean body weight between the Dynamics of Childhood Growth and Obesity Model and mean observed values in Ensanut 2012 in children from 5 to 17 years old........ 7

Supplementary table 1. Potential impact one year after the implementation of the NEDF tax on body mass index and body mass index-for-age z-score using the Dynamic Childhood Growth and Obesity Model[a,b].

	BMI (mean ± SE)			BMI-for-age z-score (mean ± SE)		
	Without tax	With tax	Difference	Without tax	With tax	Difference
**Total**	19.8±0.10	19.6±0.10	-0.19±0.01	0.5±0.03	0.4±0.03	-0.09±0.01
**Age groups**						
School-aged children	18.3±0.10	18.0±0.10	-0.20±0.01	0.53±0.04	0.42±0.04	-0.10±0.01
Adolescent	22.4±0.20	22.2±0.20	-0.17±0.01	0.47±0.05	0.41±0.06	-0.06±0.01
**Sex**						
Male	19.7±0.16	19.4±0.16	-0.20±0.01	0.51±0.04	0.40±0.05	-0.10±0.01
Female	20.0±0.10	19.8±0.15	-0.19±0.01	0.51±0.03	0.43±0.04	-0.08±0.01
**Socioeconomic status**						
Low	19.8±0.19	19.4±0.19	-0.32±0.01	0.57±0.04	0.41±0.05	-0.15±0.01
Medium	19.6±0.17	19.4±0.17	-0.24±0.01	0.46±0.05	0.35±0.05	-0.11±0.01
High	20.8±0.22	20.8±0.22	0	0.48±0.06	0.48±0.06	0

[a]The Dynamic Childhood Growth and Obesity Model (DCGO) was used to obtain two scenarios of body weight in the sample with a one-year simulation time frame. The first scenario assumed no intervention (Without tax); thus, this model only considers weight changes due to growth. The second scenario was simulated using the same inputs as scenario 1, but under the effect of the NEDF tax (With tax). Absolut differences between both scenarios represent the potential effect of NEDF tax on the prevalences of overweight or obesity at the end of the simulation year.

[b]Simulated body weight after 1 year of the NEDF tax, assumed an average reduction of 5.1% in Nonessential Energy Dense Food (NEDF) consumption at the individual level. Differences by socioeconomical status, assumed reductions of 10.2%, 5.8%, and 0% for low, medium, and high SES, respectively [15].

BMI, Body Mass Index; NEDF, nonessential energy-dense foods; SE, Standard error; p.p., percentage points.

Supplementary weight change model specification

Childhood Growth and Obesity Model

We used the Dynamics of Childhood Growth and Obesity model (DCGO) developed by Hall et al. (1) to simulate weight changes on Mexican children. The DCGO model was previously adapted to the Mexican population by Torres-Álvarez et al. to analyze the impact of the sugar sweetened beverages tax in Mexican Children (2). Briefly, the DCGO model considers the interaction between fat mass (FM), fat free mass (FFM), energy intake function (I) and energy expenditure function (E), adjusted by a body-growth term. Body weight (BW) is the result of the sum of FM and FFM which are determined by a system of ordinary differential equations. A detailed description of the equations and model implementation can be found elsewhere (1, 2). The addition of a sex-specific growth term in the DCGO model, which combines an increase on energy intake with time and an age dependent function representing the effect of complex physiological processes, allows the DGCO model to accurately simulate healthy growth and also the development of childhood obesity over time.

Model simulations

Change in weight

The DCGO model considers body weight (BW) as a function of time (t) and depends on characteristics of individual level data of children like sex (Sex), initial fat mass ($FM(0)$), initial fat free mass ($FFM(0)$), and energy intake ($I(t)$). To initialize the model we obtained sex, age, weight and height at $t = 0$ from ENSANUT 2012 survey, and calculated initial FFM and FM for each individual k using the following equations from Deurenberg et al. (3):

$$FM_k(0) = BF\%_k(0) * BW_k(0),$$

$$FFM_k(0) = BW_k(0) - FM_k(0),$$

where $BF\%_k(0)$ represents the individual's body fat percent derived as:

$$BF\%_k(0) = 1.51 * BMI_k(0) - 0.70 * age_k(0) - 3.6 * sex_k\,(1 = male, 0 = female) + 1.4$$

Then, we estimated energy intake for every individual k in ENSANUT 2012 using the DCGO model equation for reference energy intake ($I_k^{reference}(t)$). Finally, we simulated the one-year body weight ($t = 1$) for each individual k which can be represented as follows:

$$BW_k^{no\ intervention}(t) = \mathrm{BW}(\mathrm{t};\ \mathrm{Sex}_k,\ FM_k(0), FFM_k(0), I_k^{reference}(t)) =$$

To obtain the corresponding simulated body weight under the effect of the tax to nonessential energy-dense foods (NEDF), the input for energy intake was considered as:

$$I_k^{intervention}(t) = I_k^{reference}(t) - NEDF\ consumption * tax\ effect * (1 - compensation\ rate),$$

where $NEDF\ consumption$ represents the caloric intake from nonessential energy-dense foods, $tax\ effect$ corresponds to 0.102 for low socio-economic status and 0.058 for medium socio-economic status and $compensation\ rate$ equals to 0 for the main scenario with no energy compensation and 0.1 and 0.2 for the sensitivity analysis.
The one-year simulated body weight under the NEDF tax effect was computed using:

$$BW_k^{intervention}(t) = \mathrm{BW}(\mathrm{t};\ \mathrm{Sex}_k, \quad FM_k(0), FFM_k(0), I_k^{intervention}(t))$$

Finally, the change in weight for each individual k ($\Delta BW_k(t)$) is calculated as:

$$\Delta BW_k(t) = BW_k^{no\ intervention}(t) - BW_k^{intervention}(t).$$

Change in body mass index
To obtain the expected change in body mass index $BMI_k(t)$ for each individual k, we used Equation:

$$BMI_k(t) = BW_k(t)/(H_k(t))^2$$

where $BW_k(t)$ represents the estimated individual's body weight (kg) with the DCGO model, t stands for the number of days after the intervention ($t = 1$), and $H_k(t)$ represents individual's height in meters, which was estimated using growth projections using the height-for-age z-scores from WHO (4).

Change in obesity prevalence
We classified each individual's $BMI_k(t)$ into BMI categories using the WHO's BMI-for-age z-scores (4). We introduced a dummy variable $BMIcat_k(t)$(1 = obesity, 0 = normal) and calculated the change in obesity prevalence ($\Delta BMIcat_k(t)$) with Equation:

$$\Delta BMIcat_k(t) = BMIcat_k(0) - BMIcat_k(t),$$

where k represents each individual in the sample, $BMIcat_k(0)$ corresponds to the baseline BMI category ($t = 0$) and $BMIcat_k(t)$ represents the new BMI category after one-year of the NEDF tax ($t = 1$).

Dynamics of Childhood Growth and Obesity Model: validation according to nutritional status.

To validate the body weight simulations, we compared the observed average body weight according to nutritional status of children (ages 6-18) from ENSANUT 2012, with the average one-year simulated body weight without intervention from the DCGO model estimates, using data from ENSANUT 2012 (children ages 5-17) as input. Figures 1 and 2 show that our one-year predictions were consistent with the observed average weights by nutritional status for the corresponding ages in the ENSANUT survey, with average errors of 1.87 kg in underweight status, 0.73 kg in normal weight status, 0.98 kg in overweight status, 1.9 kg in obesity status and an overall error of 0.94 kg.

Supplementary figure 1. Comparison of mean body weight between the Dynamics of Childhood Growth and Obesity Model and mean observed values in Ensanut 2012 according to nutritional status in children from 5 to 17 years old.

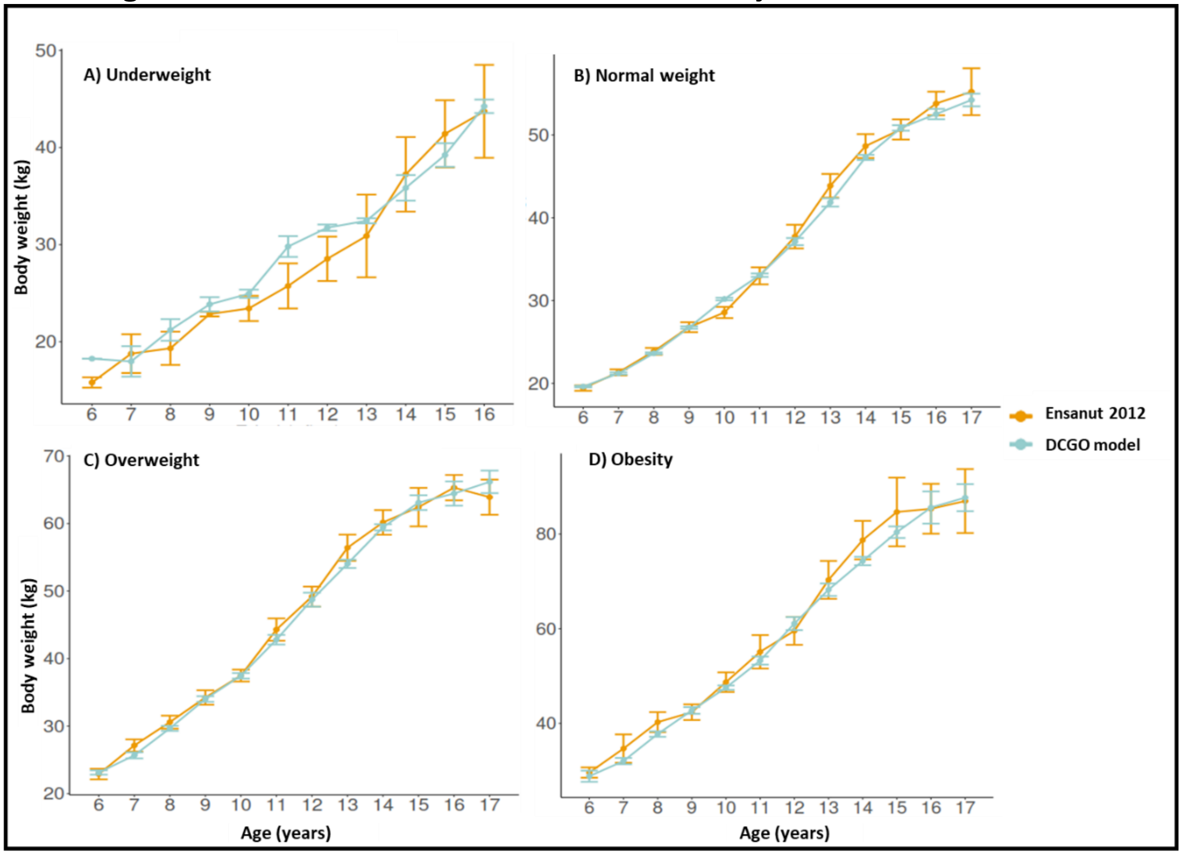

Each graph represents a comparison of body weight between the DCGO model and the observed values in Ensanut 2012 for each nutritional status: A) underweight, B) normal weight, C) overweight, and D) obese. DCGO, Dynamics of Childhood Growth and Obesity Model. Nutrition status categories are based on the standard references proposed by the World Health Organization (4).

Supplementary Figure 2. Comparison of mean body weight between the Dynamics of Childhood Growth and Obesity Model and observed mean values in Ensanut 2012 in children from 5 to 17 years old.

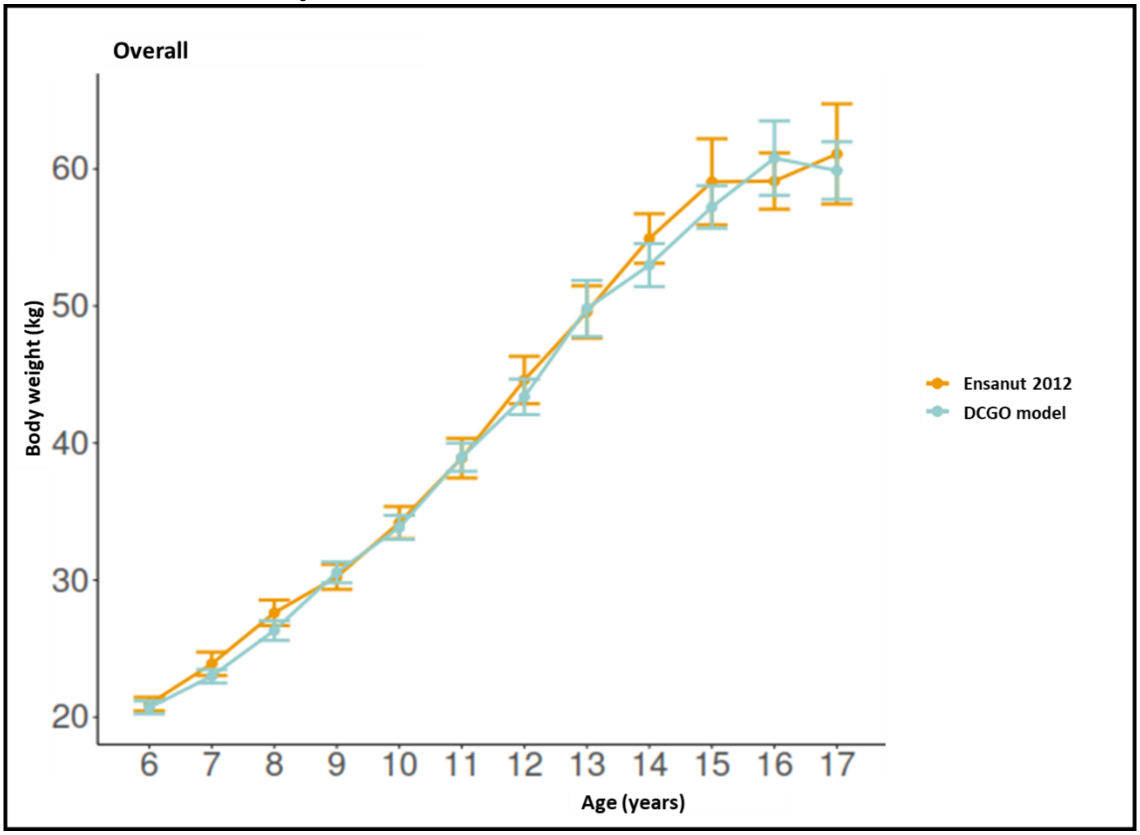

This graph represents a comparison between the simulated body weight obtained with the Dynamic Childhood Growth and Obesity Model and the observed body weight in ENSANUT 2012 between 6 and 17 years old.

References

1. Hall KD, Butte NF, Swinburn BA, Chow CC. Dynamics of childhood growth and obesity: development and validation of a quantitative mathematical model. *Lancet Diabetes Endocrinol* (2013) 1(2):97-105. Epub 2013/12/19. doi: 10.1016/S2213-8587(13)70051-2.
2. Torres-Álvarez R, Barrán-Zubaran R, Canto-Osorio F, Sánchez-Romero L, Camacho-García-Formentí D, Popkin B, et al. Body weight impact of the sugar-sweetened beverages tax in Mexican children: A modeling study. *Pediatric obesity* (2020):e12636-e. doi: 10.1111/ijpo.12636.
3. Deurenberg P, Weststrate JA, Seidell JC. Body mass index as a measure of body fatness: age-and sex-specific prediction formulas. *Br J Nutr* (1991) 65(2):105-14. doi: 10.1079/BJN19910073.
4. De Onis M. WHO child growth standards: Methods and development - Length/Height-for-age, Weight-for-age, Weight-for-length, Weight-for-height and Body mass index-for-age (2006). Available from: https://www.who.int/childgrowth/standards/Technical_report.pdf?ua=1.
